# Reliability and Validity of the Lowenstein Communication Scale

**DOI:** 10.3390/neurolint17080116

**Published:** 2025-07-29

**Authors:** Anna Oksamitni, Hiela Lehrer, Ilana Gelernter, Michal Scharf, Lilach Front, Olga Bendit-Goldenberg, Amiram Catz, Elena Aidinoff

**Affiliations:** 1 The Intensive Care for Consciousness Rehabilitation Department, the Department of Spinal Rehabilitation, Loewenstein Rehabilitation Medical Center, Raanana 4355840, Israel; annado@clalit.org.il (A.O.); hielal@clalit.org.il (H.L.); lilachf@clalit.org.il (L.F.); olgabendit@gmail.com (O.B.-G.); aidinofe@clalit.org.il (E.A.); 2 The Department of Rehabilitation, the School of Mathematics, Tel Aviv University, Tel Aviv 6139001, Israel; stat61@tauex.tau.ac.il

**Keywords:** minimally conscious state, unresponsiveness wakefulness syndrome, consciousness disorders, reliability, validity, assessment of communication

## Abstract

Background/Objectives: The Lowenstein Communication Scale (LCS) is a tool for the evaluation of communicative performance in patients with disorders of consciousness (DOC). This study investigated the reliability and validity of the LCS. Methods: We evaluated 23 inpatients with unresponsive wakefulness syndrome (UWS) and 18 in a minimally conscious state (MCS), at admission to a Consciousness Rehabilitation Department and one month later. The evaluations included assessments of LCS by two raters, and of the Coma Recovery Scale–Revised (CRS-R) by one rater. Results: Total inter-rater agreement in LCS task scoring was found in 58–100% of the patients. Cohen’s kappa values were >0.6 for most tasks. High correlations were found between the two raters on total scores and most subscales (r = 0.599–1.000, *p* < 0.001), and the differences between them were small. LCS subscales and total score intraclass correlations (ICC) were high. Internal consistency was acceptable (Cronbach’s α > 0.7) for most LCS subscales and total scores. Moderate to strong correlations were found between LCS and CRS-R scores (r = 0.554–0.949, *p* < 0.05), and the difference in responsiveness between LCS and CRS-R was non-significant. Conclusions: The findings indicate that the LCS is reliable and valid, making it a valuable clinical and research assessment tool for patients with DOC.

## 1. Introduction

Progress in critical care has led to an increase in the number of patients who survive severe traumatic and non-traumatic brain injury (TBI and NTBI). This has resulted in an increase in the number of patients with prolonged disorders of consciousness (DOC) of more than 4 weeks after the onset of brain lesion, such as unresponsive wakefulness syndrome (UWS = vegetative state = VS) and minimally conscious state (MCS) [1,2,3]. Patients with UWS demonstrate sleep and wake cycles and may maintain cortical functions associated with awareness [2], but are otherwise unresponsive to their external environment [4]. MCS involves inconsistent but clear and reproducible signs of awareness [3]. Patients in MCS show non-reflexive, purposeful behaviors, but are unable to communicate effectively [3].

The care of patients with DOC requires the assessment of different aspects of the brain injury and of the clinical condition of these patients. Quantitative assessments can provide objective data that help caregivers understand patients’ abilities, track changes over time, and tailor interventions effectively. Any sign of communicative ability is highly significant, as it offers insights into the regaining of consciousness and the emergence of elementary cognitive and communicative potential, indicating the possibility of further rehabilitation [5].

Various assessment tools have been suggested to evaluate different aspects of brain lesions. Some of them, like the Glasgow Coma Scale (GCS) and the Coma/Near-Coma (C/NC) scale, were designed for severe disabilities in acute care settings. The GCS examines patients in coma, focusing primarily on detecting conditions relevant to the acute phase, and has a prognostic value [6,7]. The C/NC was intended to evaluate patients in UWS or near-UWS, not emergence from DOC [8,9]. Other scales were intended for later stages following brain injury. These included the Wessex Head Injury Matrix (WHIM) and the Sensory Modality Assessment and Rehabilitation Technique (SMART). WHIM is a 62-item hierarchical scale for the assessment of basic behaviors, social interaction, communication, attention, and cognitive skills. It provides a total score but no guidelines for interpretation [8,10]. SMART provides guidelines to differentiate UWS from MCS and predicts recovery from UWS but requires about three weeks to complete [8,10,11]. The Disability Rating Scale (DRS) is designed to evaluate a range of performances relevant to patients with brain injuries. It consists of eight items, assessing conditions ranging from arousal and awareness to functioning in society. Scores range from 0 (no disability) to 29 (severe vegetative state) but it is not used for differential diagnosis between disorders of consciousness [12,13]. Today, the most recommended and frequently used scale for the assessment of DOC behavioral characteristics, and for distinguishing MCS from UWS and emerging from MCS, is the Coma Recovery Scale–Revised (CRS-R), with scores ranging between 0 and 23 [1,8,14,15].

Consciousness may also be indirectly detected by neuroimaging findings that showed a relationship with consciousness [15,16]. These include positron emission computed tomography (PET), electroencephalography (EEG), functional magnetic resonance imaging (fMRI), and functional near-infrared spectroscopy (fNIRS) [14,15,16]. Neuroimaging, which may pre-clinically distinguish consciousness recovery, is recommended in cases of uncertainty about evidence of consciousness, when factors interfering with neurobehavioral assessment are identified or in the absence of signs of consciousness at the bedside [16,17].

The care of patients who suffer from severe communicative disability and the monitoring of their progress require quantitative assessment of their communicative performance [17]. The Lowenstein Communication Scale (LCS) was developed primarily for such an assessment [18]. The LCS consists of 25 tasks (or items) equally divided between 5 subscales: mobility, respiration, visual responsiveness, auditory comprehension, and communication (verbal or alternative). Each task is scored 0–4 (0 = No response; 1 = One or very few signs of response; 2 = Inconsistent minimal responsiveness; 3 = Diminished response; 4 = Consistent response). The total score ranges between 0 and 100. The full LCS scoring sheet and instructions appear in Borer-Alafi et al. [18]. The original LCS version is in Hebrew (Supplementary Materials Table S1) and was translated into English using the translation and back-translation method.

In a subgroup of 22 patients with UWS and MCS after TBI, Borer-Alafi et al. found that LCS has a good inter-rater agreement [18]. They also showed that among 42 such patients, 27, who eventually showed sufficient cognitive and sub-language behaviors to be referred for further comprehensive rehabilitation, during their stay in intensive care for consciousness rehabilitation (ICCR), had significantly higher LCS scores on the motor, visual, and auditory subscales, as well as in the total scores [18]. Expert consensus indicated that the LCS has acceptable content validity [8]. Members of the interdisciplinary team at the ICCR department reported that by evaluating oral and alternative communication using the scale, they were able to identify initial signs of recovery of communication and provide means for patients to improve communication at an early stage. Speech and language clinicians with no previous experience with LCS reported that the instructions, provided in a separate manual, were clear even for first-time users. LCS seems, therefore, to be a promising tool for the evaluation of communicative performance in ICCR patients, which may assist clinicians in formulating early, targeted treatment plans.

Some of the arguments in favor of LCS use were subjective, however, and analyses of the psychometric properties of LCS did not include NTBI patients or discrimination between patients with an initial diagnosis of MCS and UWS. In addition, the expert consensus that supported LCS content validity indicated that the evidence for LCS reliability and validity was insufficient [8]. To provide the missing evidence, in this study, we further investigated LCS reliability and validity on a group of TBI and NTBI patients with DOC in ICCR, and separately on patients in MCS or UWS.

## 2. Materials and Methods

### 2.1. Study Population

We screened patients who were admitted consecutively to the ICCR Department at a Rehabilitation Medical Center between 2019 and 2021, having been transferred from general hospitals at different periods after brain lesion onset, usually within 2 weeks to 6 months from TBI or NTBI. In the ICCR Department, they received intensive care to prevent medical complications, optimize their physical condition, and enable optimum functioning and communication. The length of stay in the department is usually 2–6 months. The treatment includes medical care by physiatrists with neurosurgical consultation; nursing care; physical, occupational, communication, and music therapy; dietary, psychological, and social counseling; medication and environmental stimuli to promote arousal and communication; measures to promote feeding; and the adjustment of adaptive devices, such as wheelchairs. Patients are closely monitored for new opportunities for communication, and the ICCR staff caring for the patients also support and advise their families. Inclusion criteria were being aged between 18 and 75 years and having a clinical diagnosis of UWS or MCS at least 28 days following TBI or NTBI. Exclusion criteria were missing appropriate LCS or CRS-R data or nonconformity of the admission CRS-R scoring with a DOC diagnosis.

### 2.2. Procedure

Two raters (M.S. and G.C., referred to as raters A and B, respectively) administered the LCS (Hebrew version, (Supplementary Materials Table S1) to the patients included in the study at admission to the ICCR department (evaluation 1) and a month later (evaluation 2). The raters, who were professional speech–language pathologists, experienced in the care of DOC and LCS administration, evaluated the patients independently, each one blinded to the scoring of the other rater. The two raters performed each patient evaluation within 48 h of each other. Raters assessed the LCS within 7 days of a CRS-R assessment, administered by an occupational therapist who received training in CRS-R administration. Data collection was prospective, but for 3 patients, an occupational therapist scored a “proxy CRS-R”, not by direct observation but based on a description of patient performance, which a team member recorded, within the time frame of the study.

### 2.3. Ethical Considerations

The study was approved by the local ethics committee (identifier 0023–18-LOE). We followed the ethical principles of the Declaration of Helsinki. Informed consent was waived because the LCS is a routine evaluation in the department, but the legal guardians of the patients were informed about the study, the evaluations being performed, and their meaning.

### 2.4. Statistical Analysis

Analyses were performed for the entire group of participants with available data and, when appropriate, separately for the UWS and MCS patients. The fit of LCS total and subscale scores to normal distribution was evaluated with the Kolmogorov–Smirnov test. The differences in LCS scores between UWS, MCS, and conscious patients (those who regained consciousness during rehabilitation) were evaluated using analysis of variance (ANOVA). For the assessment of inter-rater reliability, we calculated the total agreement between the scores of raters A and B for LCS items and the corresponding Cohen’s kappa. We also examined the correlations and the differences between the subscales and total scores obtained by the two raters, using Spearman’s test and a paired sample t-test. We used intraclass correlation (ICC) to determine the inter-rater reliability of the subscales and total LCS scores. The internal consistency of the tasks within subscales and of the subscales within the total score was evaluated using Cronbach’s alpha coefficient (α). A value of Cronbach’s alpha after the elimination of an item from the analysis (α *) which is lower than α indicates that the item contributes to increasing the internal consistency of the subscale, and vice versa [19,20]. The responsiveness or sensitivity to change in communicative performance between evaluations 1 and 2, was calculated using the McNemar–Bowker test for LCS and CRS-R and compared between corresponding subscales of the scales. We used Spearman’s correlation test to calculate the correlation between LCS and CRS-R total scores, to evaluate the criterion validity of LCS. The relation between total LCS score at evaluation 1 and a diagnosis of full consciousness based on CRS-R scores at evaluation 2 was investigated using a logistic regression.

Kappa values < 0.4, 0.4–0.6, 0.6–0.8, or >0.8 indicate poor, fair-to-moderate, good, or excellent agreement, respectively [21]. Cronbach’s alpha values > 0.7 are considered acceptable [20]. ICC coefficients < 0.5, 0.5–0.75, 0.75–0.9 and >0.9 indicate poor, moderate, good, and excellent reliability, respectively [22]. Correlation coefficients of 0–±0.3 were considered weak; of ±0.3–±0.7, moderate; and ±0.7–±1, strong [23]. We considered *p*-values smaller than 0.05 to be significant. Statistical analyses were performed using the IBM Statistical Package for the Social Sciences (SPSS version 29.0.1.0).

## 3. Results

### 3.1. Participants

Of the 174 patients who were admitted to the ICCR Department between 2019 and 2021, 56 were recruited for the study based on the inclusion and exclusion criteria. Of these, 15 were further excluded: one who regained consciousness before the first evaluation, five who were recruited by mistake (two who did not meet the age criterion and three who did not have MCS or UWS for at least 28 days), and nine whose LCS or corresponding CRS-R evaluations were missing. The patients who were excluded did not significantly differ from those who were included in age (median 56, range: 17–79 and median 52, range: 20–71, respectively, *p* = 0.26), sex (80% and 68% men, respectively, *p* = 0.31), and etiology (TBI for 60% of the excluded and 56% of the included, *p* = 0.52). Table 1 presents the demographic and clinical data of the 41 remaining patients who were included in the study. Of the included participants, 22 (54%) had only LCS evaluation 1, one (2%) had only LCS evaluation 2, and 18 (44%) had both LCS evaluations completed. Based on CRS-R scores, consciousness state was UWS in 23 (56%) of the participants and MCS in 18 (44%) at evaluation 1 (admission). At evaluation 2, it was UWS in 8 of the 19 assessed patients, MCS in 3, and 8 regained full consciousness. Personal patient data are presented as Supplementary Materials Table S2).

### 3.2. LCS Scores

At evaluation 1, the mean LCS score for the entire patient population was 23 (SD = 15, range 8–66.5). For patients with UWS, it was 14 (SD = 5, range 8–27), and for those with MCS of 35 (SD = 14, range 13–67). At evaluation 2, the mean score for the entire patient population was 38 (SD = 26, range 11–98). It was 16 (SD = 5, range 11–24) for patients with UWS, 29 (SD = 11, range 18–39) for patients with MCS, and 64 (SD = 18, range 43–98) for patients who regained consciousness. At both evaluations, significant differences in LCS scores were found between the persons with different consciousness states (*p* < 0.001) but not between the two raters.

### 3.3. Inter-Rater Reliability

#### 3.3.1. Agreement in Item Scores Between Raters

Table 2 and Table 3 present the total agreement and kappa values for each LCS item, within each subscale, for all the patients, and separately for the UWS and MCS patients. For the entire patient group, total agreement in LCS scores between raters A and B was found in 59–100% of the examined patients in the different tasks at evaluation 1, and in 72–100% at evaluation 2. The total agreement was found for 80% of the patients or more, in 20 of 25 tasks. The corresponding Cohen’s kappa values were significant for all tasks, at both evaluations, 1 and 2 (*p* < 0.001). Inter-rater agreement was good to excellent (kappa values > 0.6) for all tasks, except “responsiveness”, “response to noise”, “respiration for speech production”, and “blink reflex, rhythm/fluency or speed”, at evaluation 1, which showed fair-to-moderate agreement (kappa values = 0.4–0.6).

#### 3.3.2. Correlation Between Raters in Subscale and Total Scores

The coefficients of the correlations between the scores of the paired raters for the five subscales and for the total LCS scores, at evaluations 1 and 2, were strong for the entire patient group, moderate to strong for the UWS group, and strong for most subscales of the MCS group (*p* < 0.01, Table 4 and Table 5). Only for the mobility subscale of the MCS group at evaluation 2, the correlation was not significant.

#### 3.3.3. Difference Between Raters in Subscale and Total Scores

In the subscales, we observed significant inter-rater differences at evaluation 1 only for “auditory comprehension” in the entire patient group and the UWS group, and at evaluation 2 in the entire patient group and the MCS group (*p* < 0.05). The difference resulted mainly from inter-rater differences in the “response to noise” task, which was small (0.42 points). There was a significant difference between raters in the “mobility” subscale for patients with MCS (*p* = 0.041), mainly because of differences in “responsiveness” (0.38 points) and “speech/swallow” tasks (0.31 points). There were no significant differences between raters in other subscales in the entire patient group or in the separate UWS and MCS groups (Table 4 and Table 5). In the total scores, at evaluation 1, we found a small but significant difference between rater A and B only in the entire patient group (1 point difference on a 100-point scale, *p* = 0.042, Table 4). At evaluation 2, the total scores did not differ significantly between raters (Table 5).

#### 3.3.4. Intra-Class Correlation in Subscale and Total Scores

ICC values were excellent for the total scores and the subscales but moderate for the “communication” subscale for patients with MCS and good for most of the subscale for patients with UWS (Table 6).

### 3.4. Internal Consistency

In the entire patient group, Cronbach’s alpha values of the total LCS scores were acceptable (Table 7). Cronbach’s alpha decreased when the “mobility”, “visual responsiveness”, “auditory comprehension”, and “communication” subscales were eliminated. At the same time, the elimination of the “respiration” subscale increased Cronbach’s alpha values (Table 7). In the subscales, Cronbach’s alpha values were below adequate only for “respiration.” Elimination of the majority of the tasks decreased the internal consistency of their corresponding subscales, but elimination of several tasks increased the internal consistency of their subscales (Table 7). These tasks were “assisted respiration”, “spontaneous respiration”, “matching tasks”, and “comprehension of two-step commands” at evaluation 1, and “matching tasks” at evaluation 2. Table 8 and Table 9 display the internal consistency separately for the UWS and the MCS groups.

### 3.5. Responsiveness

In 64 pairs of evaluations in which rater A assessed the 4 comparable subscales in 16 patients, LCS identified 45 score changes between evaluations 1 and 2, and CRS-R identified 38 changes. In 60 pairs of evaluations, in which rater B assessed the 4 subscales in 15 patients, LCS identified 46 score changes and CRS-R 35. The difference in responsiveness between the two scales did not reach statistical significance, however (*p* > 0.1, Table 10).

### 3.6. LCS and CRS-R Relationship

The coefficients of the correlations found between LCS and CRS-R total scores were 0.842 or above (strong) for the entire patient group (*p* < 0.001). For the first and second raters, and for evaluations 1 and 2, respectively, they equaled 0.842 and 0.843, and 0.936 and 0.949 for the entire patient group (strong); 0.582 and 0.651, and 0.914 and 0.872 for the MCS group (moderate to strong); and 0.554 and 0.601, and 0.789 and 0.853, for the UWS group (moderate to strong) (*p* < 0.05). Total LCS scores at evaluation 1 were significantly related to a diagnosis of full consciousness based on CRS-R scores at evaluation 2 (Odds ratio, OR = 1.260; 95% confidence interval, CI = 1.002–1.583; *p* = 0.048).

## 4. Discussion

This study adds evidence required to establish the suitability of LCS for bedside assessment of communicative performance in patients with DOC. It presents the only new evidence about LCS, after the original presentation of the tool [18]. It provides a more detailed demonstration of the LCS psychometric properties and allows their generalization for patients with traumatic or non-traumatic brain injuries in both UWS and MCS. The results support the use of LCS for the assessment of DOC patients in clinical or research settings, in accordance with the criteria of the American Congress of Rehabilitation Medicine [8].

The findings show that the instrument is reliable and responsive, and support its validity for the entire DOC group and each of its UWS and MCS subgroups. For all the DOC groups, LCS reliability was manifest in the good or excellent inter-rater agreement for the majority of the tasks (Table 2 and Table 3); in the generally good correlations between scores of the two raters, with only small differences between them (Table 4 and Table 5); and in the high ICC values (Table 6). It was also manifest in the good or high internal consistency values for the total LCS score and most LCS subscales (Table 7, Table 8 and Table 9). LCS responsiveness was demonstrated by the comparison showing that it was at least as responsive as CRS-R (Table 10). Together with the moderate-to-strong correlations found between the LCS and CRS-R scores, this supports LCS criterion validity.

Overall reliability was good despite the tendency of the “respiration” LCS subscale and certain tasks to compromise it. The “respiration” subscale reduced the internal consistency of the total LCS score (Table 7), and the “response to noise”, “blink reflex”, “responsiveness”, “matching”, “mimicry”, “assisted respiration”, “spontaneous respiration”, and “comprehension of two-step commands” tasks contributed to the relatively low inter-rater agreement, a small significant difference between raters’ scores, and reduced internal consistency of the subscales (Table 2, Table 3, Table 4, Table 5, Table 6, Table 7, Table 8 and Table 9).

The “respiration” subscale reduced the internal consistency because of the ceiling effect reflected in the scores of the “assisted respiration” and “spontaneous respiration” tasks, which were high relative to the tasks in the other subscales, probably because patients were admitted to the ICCR only after being taken off the ventilator.

The decrease in internal consistency that the “response to noise” task caused may be explained by the fact that at times, the response is subtle and may not be noticed. The “matching” and “comprehension of two-step commands” tasks reduced the internal consistency of their subscales, possibly because they were difficult for patients with DOC; therefore, many of the participants had a score of 0 and hardly correlated with other tasks in their subscales.

Comparison with CRS-R supports LCS validity. Previous publications showed that the CRS-R, which assesses similar properties, is itself a proper criterion for LCS assessments [1,8,15]. The Coma Recovery Scale (CRS) was developed in 1991 by Giacino et al. to evaluate consciousness; its revised version, CRS-R, introduced in 2004, is customarily used for differentiation between MCS and UWS with high reliability, validity, and sensitivity [1,24]. CRS-R is recognized as the most comprehensive and reliable behavioral assessment for DOC [8,15], and it has been translated into several languages [25,26,27,28,29]. Several publications showed that CRS-R detected responses indicating improved consciousness in patients with DOC better than the Disability Rating Scale (DRS), the Wessex Head Injury Matrix (WHIM), the Glasgow Coma Scale (GCS), and the Full Outline of Unresponsiveness scale (FOUR). They also showed reasonable correlations between CRS-R scores and the scores of these scales [1,27,28,30]. Although the correlation between LCS and CRS-R scores was good, it was not very high. This and the differences found between them confirm that the scoring of the scales is not identical and that they score somewhat different things. Although both scales include the two components characterizing emergence from MCS, functional interactive communication and functional use of two different objects [3], CRS-R scores consciousness, whereas the LCS focuses more on communication. Additional support for LCS validity is its predictive ability, demonstrated by the significant relationship between the first LCS assessment and full consciousness diagnosis at evaluation 2. Its good reliability, the supported validity, and ease of administration make the LCS suitable for assessment of communicative performance in patients with DOC. In our studies of patients with DOC, therefore, we used CRS-R for the assessment of consciousness state [31,32] and the LCS for the assessment of communication [32]. Another aspect regarding the use of the LCS is that the LCS instructions, although clear, leave some details to the clinician’s professional judgment, which likely increases the possibility of a more complete assessment of responses but may also reduce exact reproducibility.

## 5. Limitations

A limitation of the comparison between the LCS and CRS-R is that it included only subscales in which the content is common to both scales. The “respiration” subscale is not part of CRS-R, and arousal and oromotor function are not part of the LCS. These subscales, however, represent a minority of the assessed functions. Another limitation is the inability of the statistical methods we used to calculate certain measures in the absence of score variance between the examined patients, although the invariant scoring may reflect a real finding. Such invariance occurred when most of the patients achieved the maximum or the minimum score in a certain task (Table 2, Table 3, Table 4, Table 8 and Table 9). An additional limitation is the potential inaccuracy of DOC diagnosis, which we based on only one CRS-R assessment in each evaluation, whereas the patients’ consciousness level may fluctuate. The collection of data that may fluctuate on different days may have also affected CRS-R assessment and inter-rater reliability. We believe, however, that these inaccuracies were negligible because in most cases raters collected LCS data on the same day, potential deviations were offset by the number of subjects, and the close clinical observation confirmed the diagnoses. The possibility of aphasia could also have affected the CRS-R and the reliability of DOC diagnosis in patients with left hemispheric stroke (Table S2). We believe, however, that the likelihood of this is small because there was no clear diagnosis of aphasia in the patients who regained consciousness, and aphasia was suspected in only one of them. In addition, the LCS does not allow the differentiation of MCS− from MCS+. The LCS, however, was not designed to replace CRS-R in diagnosing subtle changes in consciousness but it is helpful in diagnosing the communicative performance required to determine treatment objectives in DOC patients.

## 6. Conclusions

The findings of the present study support the use of the LCS as a reliable and valid tool for assessing communication in patients with DOC after TBI and NTBI, including UWS and MCS. These findings support the ability of the LCS to accurately assess communicative performance, making it a valuable addition to the clinical and research assessment tools for patients with DOC.

## Figures and Tables

**Table 1:** Patient characteristics.

Characteristic	Value
Number	41
Age (years), median (range)	52 (20–71)
Days from injury to admission, median (range)	39 (26–103)
LOS (days), median (range)	72 (19–173)
Sex (number)	
Males	28
Females	13
Etiology (number)	
TBI	23
NTBI	18
Consciousness state at admission (number)	
UWS	23
MCS	18

Abbreviations: LOS = length of stay in intensive care for consciousness rehabilitation; TBI = traumatic brain injury; NTBI = non-traumatic brain injury; SD = standard deviation; UWS = unresponsive wakefulness syndrome; MCS = minimally conscious state.

**Table 2:** Total agreement (%) and kappa coefficients of LCS task scores, at evaluation 1.

	ALL		UWS		MCS	
Subscales and Tasks	Agreement	Kappa	Agreement	Kappa ^a^	Agreement	Kappa ^a^
Mobility						
Responsiveness	68%	0.590	77%	0.638	56%	0.391
Head/eye control	87%	0.815	82%	0.656	94%	0.910
Speech/swallow	79%	0.689	86%	0.709	69%	0.612
Extremity control	84%	0.784	77%	0.659	94%	0.908
Mimicry	92%	0.856	91%	0.450	94%	0.914
Respiration						
Assisted	97%	0.843	95%	0.776	100%	1.000
Spontaneous	100%	1.000	100%	NA	100%	1.000
For voice production	95%	0.648	95%	0.645	94%	0.644
For speech production	97%	0.493	100%	NA	94%	0.484
Coordinated	100%	1.000	100%	NA	100%	1.000
Visual responsiveness						
Gaze	79%	0.724	77%	0.623	81%	0.729
Blink reflex	66%	0.554	55%	0.286	81%	0.729
Environmental stimulation	92%	0.873	95%	0.651	88%	0.826
Tracking	92%	0.843	100%	NA	81%	0.755
Matching tasks	100%	1.000	100%	NA	100%	1.000
Auditory comprehension						
Response to noise	58%	0.467	55%	0.312	63%	0.455
Response to voice	92%	0.886	95%	0.867	88%	0.837
Compr 1-step command	95%	0.889	95%	0.645	94%	0.905
Object recognition	100%	1.000	100%	NA	100%	1.000
Compr 2-step command	100%	1.000	100%	NA	100%	1.000
Communication						
Articulators or assist	95%	0.779	95%	NA	94%	0.860
Basic speech or use body	95%	0.696	100%	NA	88%	0.667
Articulation or initiative	95%	0.757	100%	NA	88%	0.722
Rhythm/fluency or speed	92%	0.580	100%	NA	88%	0.549
Message quality	97%	0.790	100%	NA	94%	0.755

Notes: ^a^ Kappa calculations were not available for tasks with uniform or nearly uniform scores. Abbreviations: NA = not available; Coordinated = coordinated for speech; Compr = comprehension; Articulators or assist = use of speech articulators or need for outside assistance; Basic speech or use body = basic speech or use of body parts.

**Table 3:** Total agreement (%) and kappa coefficients of LCS cores, at evaluation 2.

	ALL		UWS		MCS	
Subscales and Tasks	Agreement	Kappa	Agreement	Kappa ^a^	Agreement	Kappa ^a^
Mobility						
Responsiveness	78%	0.692	70%	0.538	88%	0.600
Head/eye control	78%	0.710	90%	0.859	63%	0.226
Speech/swallow	94%	0.923	100%	1.000	88%	0.619
Extremity control	78%	0.706	100%	1.000	50%	0.304
Mimicry	72%	0.650	80%	0.697	63%	0.529
Respiration						
Assisted	100%	NA	100%	NA	100%	NA
Spontaneous	100%	NA	100%	NA	100%	NA
For voice production	83%	0.684	90%	0.615	75%	0.644
For speech production	83%	0.640	90%	NA	75%	0.660
Coordinated	94%	0.778	100%	NA	88%	0.742
Visual responsiveness						
Gaze	89%	0.854	90%	0.855	88%	0.619
Blink reflex	83%	0.745	80%	0.740	88%	0.467
Environmental stimulation	89%	0.841	100%	1.000	75%	0.619
Tracking	78%	0.684	90%	0.778	63%	0.478
Matching tasks	100%	1.000	100%	1.000	100%	1.000
Auditory comprehension						
Response to noise	78%	0.701	60%	0.500	100%	1.000
Response to voice	89%	0.847	100%	1.000	75%	0.556
Compr 1-step command	89%	0.836	100%	1.000	75%	0.667
Object recognition	89%	0.789	90%	0.615	88%	0.833
Compr 2-step command	100%	1.000	100%	NA	100%	1.000
Communication						
Articulators or assist	100%	1.000	100%	1.000	100%	1.000
Basic speech or use body	89%	0.737	90%	NA	88%	0.818
Articulation or initiative	83%	0.635	90%	NA	75%	0.644
Rhythm/fluency or speed	100%	1.000	100%	NA	100%	1.000
Message quality	89%	0.735	90%	NA	88%	0.810

Notes: ^a^ Kappa calculations were not available for tasks with uniform or nearly uniform scores.

**Table 4:** Correlation and differences between raters at evaluation 1.

LCS Subscale	Group	Rater	Mean	SD	r	*p* *	t	*p* **
Mobility	All	A	5.6	4.6	0.899	<0.001	−1.597	0.119
		B	5.9	4.7				
	UWS	A	3.0	2.4	0.714	<0.001	−0.295	0.771
		B	3.1	2.4				
	MCS	A	9.1	4.2	0.899	<0.001	−2.236	0.041
		B	9.9	4.1				
Respiration	All	A	8.0	0.8	0.828	<0.001	−1.000	0.324
		B	8.1	0.9				
	UWS	A	8.0	0.4	0.774	<0.001	0.000	1.000
		B	8.0	0.5				
	MCS	A	8.1	1.1	0.895	<0.001	−1.464	0.164
		B	8.3	1.3				
Visual responsiveness	All	A	4.7	4.8	0.910	<0.001	−0.508	0.614
		B	4.9	5.1				
	UWS	A	1.6	1.8	0.713	<0.001	0.000	1.000
		B	1.6	1.7				
	MCS	A	8.8	4.4	0.890	<0.001	−0.689	0.501
		B	9.1	4.9				
Auditory comprehension	All	A	3.4	3.8	0.898	<0.001	−2.720	0.010
		B	3.9	3.8				
	UWS	A	1.0	1.6	0.624	0.002	−2.270	0.034
		B	1.6	1.8				
	MCS	A	6.6	3.6	0.964	<0.001	−1.464	0.164
		B	7.0	3.7				
Communication	All	A	0.7	2.0	0.921	<0.001	−0.572	0.571
		B	0.7	2.1				
	UWS	A	0.05	0.21	NA ^a^	NA	1.000	0.329
		B	0.00	0.00				
	MCS	A	1.6	3.0	1.000	NA	−1.464	0.164
		B	1.7	3.0				
LCS total	All	A	22.9	14.6	0.929	<0.001	−2.099	0.042
		B	23.9	14.9				
	UWS	A	13.5	5.3	0.750	<0.001	−1.109	0.280
		B	14.2	5.0				
	MCS	A	34.3	14.1	0.930	<0.001	−1.840	0.083
		B	35.7	14.5				

Notes: ^a^ Correlation calculation was not available because of 0 variance for rater B; * = of the correlation between raters’ scores; ** = of the difference between raters’ scores. Abbreviations: LCS = Loewenstein communication scale; All = all the participating patients; r = Spearman’s correlation coefficient; t = the t statistic of the difference between raters’ scores; *p* = significance.

**Table 5:** Correlation and differences between raters at evaluation 2.

LCS Subscale	Patient Group	Rater	Mean	SD	r	*p* *	t	*p* **
Mobility	All	A	10.6	6.9	0.952	<0.001	0.345	0.734
		B	10.5	6.4				
	UWS	A	5.6	4.3	0.985	<0.001	−1.309	0.223
		B	6.0	4.4				
	MCS	A	16.9	3.4	0.595	0.120	1.342	0.222
		B	16.1	3.2				
Respiration	All	A	9.6	3.1	0.994	<0.001	−0.437	0.668
		B	9.6	3.1				
	UWS	A	8.2	0.4	1.000	NA	NA	NA
		B	8.2	0.4				
	MCS	A	11.3	4.1	0.943	<0.001	−0.424	0.685
		B	11. 4	4.2				
Visual responsiveness	All	A	9.1	7.4	0.976	<0.001	0.270	0.790
		B	9.0	7.4				
	UWS	A	4.5	5.3	0.978	<0.001	0.000	1.000
		B	4.5	5.2				
	MCS	A	14.8	5.4	0.924	0.001	0.284	0.785
		B	14.6	5.7				
Auditory comprehension	All	A	6.9	6.5	0.970	<0.001	−2.149	0.046
		B	7.4	6.2				
	UWS	A	3.1	3.4	0.826	0.003	−1.168	0.273
		B	3.6	2.5				
	MCS	A	11.6	6.4	0.988	<0.001	−2.376	0.049
		B	11.3	6.1				
Communication	All	A	3.2	5.5	0.995	<0.001	1.381	0.185
		B	2.8	5.2				
	UWS	A	0.4	1.3	1.00	NA	1.000	0.343
		B	0.1	0.3				
	MCS	A	6.8	6.7	1.000	NA	0.935	0.381
		B	6.3	6.4				
LCS total	All	A	38.1	26.3	0.986	<0.001	−0.243	0.811
		B	38.3	25.3				
	UWS	A	21.4	12.7	0.965	<0.001	−0.784	0.451
		B	22.0	11.2				
	MCS	A	61.0	22.5	0.922	0.001	0.209	0.840
		B	60.1	21.8				

Notes: * = of the correlation between raters’ scores, ** = of the difference between raters’ scores.

**Table 6:** Intraclass correlation coefficients (ICC).

LCS Subscale	Patient Group	ICC Evaluation 1	95% CI Evaluation 1	ICC Evaluation 2	95% CI Evaluation 2
Mobility	All	0.975	0.952–0.987	0.989	0.972–0.996
	UWS	0.899	0.758–0.958	0.988	0.950–0.997
	MCS	0.973	0.923–0.991	0.940	0.699–0.988
Respiration	All	0.962	0.928–0.980	0.992	0.980–0.997
	UWS	0.855	0.651–0.940	1.000	
	MCS	0.980	0.943–0.993	0.990	0.949–0.998
Visual responsiveness	All	0.974	0.950–0.986	0.996	0.991–0.999
	UWS	0.797	0.512–0.916	0.998	0.992–0.999
	MCS	0.963	0.898–0.987	0.987	0.936–0.997
Auditory comprehension	All	0.978	0.957–0.988	0.992	0.980–0.997
	UWS	0.855	0.650–0.940	0.946	0.783–0.987
	MCS	0.980	0.942–0.993	0.996	0.982–0.999
Communication	All	0.995	0.991–0.998	0.987	0.966–0.995
	UWS	NA ^a^		0.640	(−0.449)–0.911
	MCS	0.997	0.991–0.999	0.987	0.933–0.997
LCS total	All	0.989	0.980–0.994	0.995	0.986–0.998
	UWS	0.913	0.791–0.964	0.987	0.952–0.997
	MCS	0.987	0.966–0.995	0.987	0.934–0.997

Notes: ^a^ ICC was not available because lack of variance of the scores given by rater Abbreviations: CI = confidence interval.

**Table 7:** Internal consistency of LCS score in the entire patient group ^a^.

	Evaluation 1				Evaluation 2			
Subscales and Tasks	Rater A		Rater B		Rater A		Rater B	
	α	α *	α	α *	α	α *	α	α *
Mobility	0.901		0.887		0.962		0.939	
Responsiveness		0.881		0.848		0.959		0.916
Head/eye control		0.871		0.859		0.952		0.922
Speech/swallow		0.879		0.868		0.948		0.908
Extremity control		0.884		0.878		0.956		0.937
Mimicry		0.879		0.860		0.950		0.934
Respiration	0.620		0.649		0.957		0.915	
Assisted		0.736		0.747		NA		NA
Spontaneous		0.683		0.709		NA		NA
For voice production		0.330		0.429		0.935		0.817
For speech production		0.404		0.401		0.908		0.827
Coordinated		0.485		0.508		0.962		0.958
Visual responsiveness	0.902		0.910		0.955		0.952	
Gaze		0.848		0.854		0.933		0.932
Blink reflex		0.872		0.897		0. 938		0.942
Environmental stimulation		0.856		0.862		0.931		0.924
Tracking		0.874		0.883		0.934		0.926
Matching tasks		0.930		0.934		0.979		0.971
Auditory comprehension	0.828		0.783		0.925		0.918	
Response to noise		0.726		0.740		0.933		0.935
Response to voice		0.688		0.616		0.898		0.893
Compr 1-step command		0.725		0.656		0.894		0.878
Object recognition		0.826		0.787		0.889		0.875
Compr 2-step command		0.872		0.817		0.921		0.913
Communication	0.906		0.907		0.934		0.928	
Articulators or assist		0. 854		0.842		0.925		0.942
Basic speech or use body		0.911		0.923		0.924		0.904
Articulation or initiative		0.863		0.863		0.905		0.904
Rhythm/fluency or speed		0.900		0.916		0.930		0.908
Message quality		0.886		0.885		0.908		0.903
Total LCS	0.860		0.853		0.925		0.922	
Mobility		0.766		0.764		0.893		0.891
Respiration		0.898		0.893		0.942		0.931
Visual responsiveness		0.787		0.781		0.889		0.891
Auditory compr		0.758		0.749		0.884		0.887
Communication		0.865		0.851		0.913		0.909

Notes: ^a^ α calculation was not available for certain items because of the homogeneity of scores, * if the item is deleted. Abbreviations: α = Cronbach’s alpha.

**Table 8:** Internal consistency of LCS scores in UWS and MCS at evaluation 1 ^a^.

	UWS				MCS			
Subscales and Tasks	Rater A		Rater B		Rater A		Rater B	
	α	α *	α	α *	α	α *	α	α *
Mobility	0.689		0.744		0.875		0.799	
Responsiveness		0.668		0.628		0.845		0.765
Head/eye control		0.562		0.625		0.857		0.781
Speech/swallow		0.583		0.742		0.865		0.777
Extremity control		0.585		0.639		0.846		0.745
Mimicry		0.744		0.787		0.832		0.732
Respiration	0.123		0.220		0.698		0.741	
Assisted		NA		NA		0.778		0.806
Spontaneous		NA		NA		0.765		0.806
For voice production		NA		NA		0.426		0.533
For speech production		NA		NA		0.492		0.511
Coordinated		NA		NA		0.573		0.606
Visual responsiveness	0.717		0.609		0.881		0.892	
Gaze		0.463		0.273		0.815		0.826
Blink reflex		0.368		0.148		0.856		0.885
Environmental stimulation		0.823		0.718		0.837		0.851
Tracking		NA		NA		0.866		0.886
Matching tasks		NA		NA		0.893		0.891
Auditory comprehension	0.601		0.489		0.831		0.805	
Response to noise		0.684		0.750		0.773		0.765
Response to voice		0.183		0.138		0.708		0.693
Compr 1-step command		0.655		0.497		0.737		0.700
Object recognition		NA		NA		0.825		0.792
Compr 2-step command		NA		NA		0.873		0.827
Communication	NA		NA		0.902		0.891	
Articulators or assist						0.839		0.815
Basic speech or use body						0.916		0.912
Articulation or initiative						0.860		0.842
Rhythm/fluency or speed						0.896		0.901
Message quality						0.879		0.864
Total LCS	0.707		0.680		0.849		0.834	
Mobility		0.567		0.410		0.761		0.773
Respiration		0.740		0.786		0.870		0.853
Visual responsiveness		0.546		0.563		0.805		0.797
Auditory comprehension		0.496		0.483		0.762		0. 724
Communication		0.762		NA		0.848		0.819

Notes: ^a^ α calculation was not available for certain items because of the homogeneity of scores, * if the item is deleted.

**Table 9:** Internal consistency of LCS scores in UWS and MCS at evaluation 2 ^a^.

	UWS				MCS			
Subscales and Tasks	Rater A		Rater B		Rater A		Rater B	
	α	α *	α	α *	α	α *	α	α *
Mobility	0.919		0.921		0.792		0.712	
Responsiveness		0.893		0.876		0.870		0.765
Head/eye control		0.887		0.893		0.633		0.561
Speech/swallow		0.916		0.893		0.667		0.557
Extremity control		0.920		0.936		0.770		0.693
Mimicry		0.884		0.904		0.701		0.654
Respiration	NA		NA		0.961		0.914	
Assisted						NA		NA
Spontaneous						NA		NA
For voice production						0.927		0.810
For speech production						0.924		0.824
Coordinated						0.973		0.969
Visual responsiveness	0.934		0.931		0.887		0.897	
Gaze		0.893		0.892		0.863		0.886
Blink reflex		0.910		0.923		0.847		0.859
Environmental stimulation		0.894		0.889		0.828		0.841
Tracking		0.910		0.900		0.822		0.840
Matching tasks		0.966		0.960		0.946		0.939
Auditory comprehension	0.853		0.735		0.899		0.901	
Response to noise		0.900		0.750		0.923		0.927
Response to voice		0.807		0.675		0.873		0.872
Compr 1-step command		0.809		0.630		0.874		0.861
Object recognition		0.759		0.661		0.827		0.824
Compr 2-step command		NA		NA		0.867		0.892
Communication	NA		NA		0.905		0.900	
Articulators or assist		1				0.898		0.922
Basic speech or use body		1				0.896		0.869
Articulation or initiative		1				0.869		0.869
Rhythm/fluency or speed		NA				0.883		0.856
Message quality		1				0.871		0.870
Total LCS	0.825		0.772		0.887		0.884	
Mobility		0.708		0.584		0.866		0.870
Respiration		0.869		0.814		0.883		0.860
Visual responsiveness		0.713		0.603		0.842		0.843
Auditory comprehension		0.691		0.643		0.847		0.867
Communication		0.846		0.813		0.865		0.852

Notes: ^a^ α calculation was not available for certain items because of the homogeneity of scores, * if the item is deleted.

**Table 10:** Responsiveness to changes in the Loewenstein Communication Scale (LCS) and Coma Recovery Scale–Revised (CRS-R). Yes refers to the number of patients whose scores on either LCS or CRS-R changed between evaluations; No refers to the number of those whose scores did not change.

				LCS Rater A			LCS Rater B		
Subscale	Patients			No	Yes	Total	No	Yes	Total
Mobility	All	CRS-R	No	2	3	5	1	4	5
			Yes	2	9	11	0	10	10
			Total	4	12	16	1	14	15
	UWS	CRS-R	No	2	2	4	1	3	4
			Yes	2	2	4	0	4	4
			Total	4	4	8	1	7	8
	MCS	CRS-R	No	0	1	1	0	1	1
			Yes	0	7	7	0	6	6
			Total	0	8	8	0	7	7
Visual responsiveness	All	CRS-R	No	2	3	5	2	3	5
			Yes	0	11	11	0	10	10
			Total	2	14	16	2	13	15
	UWS	CRS-R	No	2	3	5	2	3	5
			Yes	0	3	3	0	3	3
			Total	2	6	8	2	6	8
	MCS	CRS-R	No	0	0	0	0	0	0
			Yes	0	8	8	0	7	7
			Total	0	8	8	0	7	7
Auditory comprehension	All	CRS-R	No	3	2	5	3	2	5
			Yes	1	10	11	0	10	10
			Total	4	12	16	3	12	15
	UWS	CRS-R	No	2	1	3	2	1	3
			Yes	0	5	5	0	5	5
			Total	2	6	8	2	6	8
	MCS	CRS-R	No	1	1	2	1	1	2
			Yes	0	6	6	0	5	5
			Total	1	7	8	1	6	7
Communication	All	CRS-R	No	9	2	11	8	2	10
			Yes	0	5	5	0	5	5
			Total	9	7	16	8	7	15
	UWS	CRS-R	No	7	1	8	7	1	8
			Yes	0	0	0	0	0	0
			Total	7	1	8	7	1	8
	MCS	CRS-R	No	2	1	3	1	1	2
			Yes	0	5	5	0	5	5
			Total	2	6	8	1	6	7

Abbreviations: LCS = changes identified by LCS; CRS-R = changes identified by CRS-R.

## Data Availability

The data that support this study will be shared upon reasonable request sent to the corresponding author.

## Supplementary Materials

The following supporting information can be downloaded at: https://www.mdpi.com/article/10.3390/neurolint17080116/s1, Table S1: Hebrew LCS form; Table S2: Individual patient data.
