# Supplementary material for: Reliability and Validity of the Lowenstein Communication Scale
Source: Neurol Int. 2025 Jul 29;17(8):116. doi: 10.3390/neurolint17080116 (PMC12388834; doi:10.3390/neurolint17080116)
Supplement: Supplementary file 1 [file neurolint-17-00116-s001.zip › Table S1 (Hebrew LCS form) and instructions 070725.pdf]

המחלקה לטיפול הפרעות בתקשורת

טופס בדיקה ביחידת טיפול נמרץ

שם הנבדק : ____________________

מפתח להערכה :

ב- % 0) לא פונקציונלי
1) במקצת פונקציונלי
2) חלקית פונקציונלי
3) על פי רוב פונקציונלי
4) פונקציונלי

ת א ר י ך			
**מוטוריקה כללית**<br>1) תגובתיות החזרית<br>2) שליטה על הראש/עיניים<br>3) שליטה על גפיים<br>4) איברי הדיבור(בליעה/ריור)<br>5) מימיקה<br>%	[]	[]	[]
**נשימה** [] **טובוס**<br>[] **טרכיאוסטומיה**<br>[] **פתולוגיה**<br>1) הנשמה<br>2) ספונטנית<br>3) להפקת קול<br>4) לדיבור<br>5) קאורדינטיבית לדיבור<br>%	[]	[]	[]
**תגובה חזותית**<br>1) מבט<br>2) החזר הגנה<br>3) תגובה לגירוי סביבתי<br>4) עקיבה<br>5) התאמה זהה<br>%	[]	[]	[]
**הבנה שמיעתית**<br>1) תגובה לרעש<br>2) תגובה לדיבור (לקול)<br>3) ביצוע פקודות (מרכיב אחד)<br>4) זיהוי פריטים<br>5) ביצוע פקודות (שני מרכיבים)<br>%	[]	[]	[]
**תקשורת**<br>**מילולית**<br>1) שימוש באיברי הדיבור<br>2) דיבור בסיסי<br>3) היגוי<br>4) קצב/רצף<br>5) איכות ההבעה<br>**חליפית**<br>1) צורך בעזרה חיצונית<br>2) שימוש באברי הגוף<br>3) יזימה<br>4) מהירות<br>5) איכות העברת המסר<br>%	[]<br>[ ]	[]<br>[ ]	[]<br>[ ]

סה"כ ב- % ____________________

ה ע ר ו ת : ____________________

הנחיות לבדיקת מ.ת.מ

אבחון תקשורתי זה לחולים במצב של תגובות מינימליות מורכב משני חלקים (מ.ת.מ.):

- דף ראשון לרישום כללי והתרשמויות ממצב החולה, בו יצויינו בראש הדף, במקומות המתאימים הפרטים האישיים של החולה (מדבקה, תאריך הפגיעה, אתיולוגיה, ואבחנה במידה ויש).
  דף זה מיועד לרישום כל מידע ו/או התרשמות שלא נכללים בעמוד השני במסגרת אחד הפרמטרים (התנהגות, דוחי משפחה/מחלקה, ישיבות צוות, התרשמות אישית של הבודק וכד') .

- הדף השני הוא טופס הבדיקה המובנית. בבדיקה זו 5 תחומים ובכל אחד מהם 5 פרמטרים תפקודיים . כל פרמטר נבדק ע"פי 5 דרגות פונקציה. הציון ניתן באחוזים מ- 0% עבור מצב לא פונקציונאלי ועד 4% עבור מצב פונקציונאלי (ראה פירוט בראש דף הבדיקה).

  הציון המקסימלי עבור כל תחום בדיקה הוא 20%, ומכאן שסה"כ הציון המקסימלי לבדיקה כולה הוא 100% .

<u>תחומי הבדיקה</u>

5 הפרמטרים המוצגים בתוך כל תחום מופיעים בסדר קושי/התפתחותי עולה כשהפרמטר השלישי ב-4 התחומים הראשונים בכל תחום עשוי להוות את שלב המעבר ממצב של חוסר הכרה למצב הכרתי מסוים .

5 תחומי הבדיקה הם :

- **מוטוריקה כללית**
- **נשימה**
- **תגובה חזותית**
- **הבנה שמיעתית**
- **תקשורת**
- **פירוט הפרמטרים בכל 5 תחומי הבדיקה**

<u>מוטוריקה כללית</u>
חמשת הפרמטרים בתחום בדיקה זו :

1. <u>תגובתיות החזרית</u> -
זוהי למעשה תצפית על החולה על מנת ללמוד את רפרטואר תגובותיו הגופניות שעל פיהם נוכל לזהות את תגובתו לגירויים שפתיים ספציפיים . אנו מתבססים בעיקר על הופעת רפלקסים נוירולוגים שונים בין אם פתולוגיים או תקינים. למשל: החזרי הגנה (Startling, Blink מורו, כאב) מימיקה ותנועות גוף. הציונים יינתנו לפי :

- 0% אין תגובה .
- 1% סימנים בודדים, מעטים של תגובה .
- 2% תגובתיות מצומצמת לא עקבית .
- 3% מגיב לגירויים אך עם דעיכה בתגובה .
- 4% מגיב עקבי.

2. <u>שליטה על הראש/עיניים</u> -
זהו האומדן לגבי יכולת החולה לשלוט על החזקת הראש, חגורת הכתפיים והגו. כמו כן אומדן זה מתייחס גם למצב תנועתיות העיניים, ומציין חסרים מוטוריים אפשריים שלהן, כגון שיתוק של עצבים II III VI , מצב העפעפיים- [פתיחה וסגירה, פטוזיס] .יש לציין סימטריה או אסימטריה במצב העיניים . הציונים יינתנו לפי :

- 0% לא שולט על החזקת הראש, אין תנועות עיניים .
- 1% אין החזקת ראש, ממצמץ או מזיז עיניים בצורה מוגבלת.
- 2% מחזיק ראש לפרקי זמן קצרים או מזיז אותו לצדדים לא עקבי, בתמיכה. מזיז עיניים לא עקבית .
- 3% מחזיק ראש לפרקי זמן ארוכים, מסוגל להזיז ראש לצדדים די בעקביות. מזיז עיניים די בעקביות .
- 4% יש תנועות ראש ו/או תנועות עיניים טובות .

3. <u>שליטה על גפיים</u> -
זהו אומדן לגבי יכולת החולה לשלוט על 4 הגפיים, יש לשים לב לקיום של פרזיס, פלגיה, פגיעות אורטופדיות, תנועות נלוות, תנועות סטריוטיפיות, פרסברטיביות ספונטניות וג'סטות מכוונות. הציונים יינתנו לפי :

- 0% אין תנועות ב-4 הגפיים (קואדרי-פלגיה) .
- 1% מעט תנועות מכוונות באחת מהגפיים לפחות.
- 2% תנועות מכוונות ברורות באחת מהגפיים לפחות לפרקי זמן קצרים או בחוסר עקביות .
- 3% תנועות מכוונות לפחות ביד אחת לפרקי זמן משמעותיים עם דעיכה אפשרית בפעולה .
- 4% תנועות מכוונות וברורות לפחות ביד אחת בעקביות .

.../.

4. איברי הדיבור (בליעה/ריור) -
יש לנסות לאמוד את מצב תנועתיות איברי הדיבור ובמידת האפשר להעביר בדיקת דיסארטריה כדי לקבוע מצב תנועתיות שרירי הפנים, הפה, והצוואר. כמו כן, חשוב להתייחס למצב המנשך (שיניים תותבות, מצב השיניים הקיימות, יכולת נשיכה, טריסמוס). הציונים יינתנו לפי :

- 0% אין תנועתיות של איברי הדיבור, אין תנועות בליעה ספונטנית , יש ריור . (לציין תנוחת הנבדק בעת הבדיקה) .
- 1% תנועות פתולוגיות : לעיסה, חריקת שיניים, דחיקת לשון, פתיחת פה מוגזמת, jaw jerk בפיהוק, טריסמוס וכו' ספונטנית או לגירוי .
- 2% תנועתיות ספונטנית לא פתולוגית(פיהוק, שיעול, גיהוק, תנועות לשון לליקוק) או לגירוי . יש לציין אם קיים פציאליס .
- 3% הפעלה חלקית של איברי הדיבור/הבליעה (ע"פי בדיקת דיסארטריה) .
- 4% הפעלה טובה של איברי דיבור/בליעה (ע"פ בדיקת דיסארטריה).

5. מימיקה -
אומדן זה משקף את יכולת החולה להביע את הלך רוחו ע"י הבעות פנים שונות. נבדקת דרגת ההתאמה בין ההבעות הנצפות לבין הגירוי (פנימי או חיצוני) : כאב, שמחה, עצב, סלידה, התנגדות, הימנעות וכד') הציונים יינתנו לפי :

- 0% אין הבעות , מבט בוהה, פנים קפואות.
- 1% שינויים מסוימים מעטים בברק העיניים או בהבעה הכללית .
- 2% שינויים בהבעה (כאב, בכי, הנאה..) לפרקי זמן קצרים, חוסר עקביות בהבעה .
- 3% שינויים ברורים בהבעה עם דעיכה .
- 4% מביע רגשות ע"י מימיקה מתאימה .

.../.

<u>נשימה :</u>
לפני בדיקת חמשת הפרמטרים בתחום זה, יש לציין האם יש לחולה: טובוס (דרך פה או אף), טרכיאוסטומיה (המצאות בועית ומצבה, T-Tube, סוג וגודל הקנולה), או פתולוגיה נשימתית אחרת או נוספת : הצרות, בצקת, פוליפ, גרנולציות, מרסה, צניחת קנה, מצב גלוטיס וכד׳. הפרטים הרלוונטיים למצבים אלו יכתבו בדף מס׳ 1 של הבדיקה בתאריך הבדיקה, ובדף מס׳ 2 יסומן הסעיף הרלוונטי בלבד.

1. <u>הנשמה</u> : הערכים הפונקציונאליים (באחוזים) ניתנים כדלקמן :

- 0% הנשמה מלאה (Bennet אוטומטי או דומה) - החולה אינו נושם בעצמו .
- 1% במהלך גמילה מהנשמה מלאה, הפסקות קצובות עפ"י הוראות רופא .
- 2% הנשמה בחלקי היממה בלבד-הפסקות גדולות ו/או עקביות יותר.
- 3% הנשמה תמיכתית בלבד ו/או תוספת חמצן או דומה ; מעקב אסטרופ.
- 4% נושם ספונטנית.

2. <u>ספונטנית</u>:

- 0% מינימלית, בסיסית לחיים, הפסקות נשימתיות (Apnea) מרובות.
- 1% שליטה נשימתית ירודה ושטחית - בקרה אסטרופ קבועה .
- 2% הפרעות בקצב הנשימה ; בדיקה אסטרופ חלקית .
- 3% חוסר עקביות, התארגנות לא מושלמת
- 4% שליטה נשימתית טובה, לחץ סובגלוטי טוב .

<u>הערה:</u> בשלושת הפרמטרים הבאים, הציון באחוזים הוא אך ורק לתפקוד הנשמתית כפי שמתבטא ביכולת הפקת הקול של הנבדק.

3. <u>להפקת קול</u> :

- 0% אין נשימה מספקת להפקת קול, אפוניה.
- 1% הפקת קולות בודדים חלשים לא עקביים
- 2% היפופוניה - אין מספיק לחץ סובגלוטי (אנחות המהום, לחשים).
- 3% דיספוניה - קול נשוף
- 4% קול בגדר הנורמה (מבחינת עוצמה) .

.../.

4. <u>לדיבור</u> :

- 0% לא מספקת לדיבור
- 1% מספקת להפקה לא עקבית או בסיסית של מילים בודדות.
- 2% מספקת לצמדי מילים או לחלק ממשפט בלבד.
- 3% הדיבור קטוע או לא יציב עקב הפרעות נשימתיות. הנשימה לא מספקת לסיום מבע של 3-4 מילים.
- 4% מספקת להפקת מבעים של 3-4 מילים באופן עקבי .

5. <u>קואורדינציה נשימתית לדיבור</u> :

- 0% אפוניה/אפרקסיה .
- 1% היפופניה חריפה, תנודות בתפקוד הקולי על רקע של שליטה נשימתית.
- 2% משך נשיפה קצר , נשיפתיות בולטת
- 3% דיספוניה, דיפלופוניה על רקע קוצר נשימה .
- 4% שליטה נשימתית תקינה לדיבור

.../.

<u>תגובה חזותית</u>

בתחום בדיקה זה ההתמקדות היא ביכולת המטופל להשתמש בערוץ החזותי, כמובן במסגרת יכולתו המוטורית/ויזואלית (פרמטר 2 במוטוריקה כללית).

1. <u>מבט</u>

- 0% אין שימוש בערוץ החזותי (עיניים עצומות, אין תנועת עיניים, אין שינוי במצב האישונים לגירוי).
- 1% מבט בוהה.
- 2% מבט ער, תנועות עיניים לכאורה מכוונות לסביבה.
- 3% ניסיונות לא עקביים בהתמקדות בגירוי.
- 4% מבט עירני עם יכולת התמקדות.

2. <u>החזר הגנה</u> (Blink)

הבדיקה מתבצעת ע"י העברה עדינה ומתונה של כף יד/אצבע הבודק את מול עיני הנבדק. יש לשים לב שהתגובה לא נגרמת ממשוב אויר וכמו כן להגביל את כמות הגירויים הניתנים בזמן נתון. מאידך, יש לערוך תצפית על אופן וכמות המצמוצים הספונטניים של הנבדק ולהבדילם מתגובת החזר ההגנה בבדיקה.

- 0% אין החזר הגנה.
- 1% מגיב לעיתים רחוקות.
- 2% חוסר עקביות בתגובות.
- 3% דעיכה בתגובות במשך זמן הבדיקה.
- 4% החזר הגנה עקבי.

3. <u>גירוי סביבתי</u>

פרמטר זה מתייחס לתגובות הנבדק מרגע כניסת הבודק, בן אדם אחר (איש צוות, משפחה וכו') או הופעת פריט כלשהו בשדה ראיתו של הנבדק. חשוב לדעת את יכולתו הויזואלית (מוטורית/סנסורית) של הנבדק לפני הבדיקה (פרמטר 2 במוטוריקה כללית).

- 0% אינו מגיב לגירוי סביבתי.
- 1% התמקדות בגירוי באופן לא עקבי ו/או דעיכה (הפרעות ב-Shifting).
- 2% תגובה איטית עד איטית מאוד לגירוי ו/או דעיכה מהירה של התגובה.
- 3% מגיב מהר לגירוי, אך מופיעה דעיכה.
- 4% מגיב לגירויים בצורה מותאמת ללא דעיכה.

.../.

4. עקיבה :

- 0% אינו עוקב .
- 1% עוקב סלקטיבית לא עקבי.
- 2% עקיבה סלקטיבית אך עקבית .
- 3% עקיבה עם דעיכה .
- 4% עקיבה טובה ועקבית .

5. התאמה זהה :
חומרי הבדיקה :סטים זהים של תמונות או פריטים יומיומיים (לדוג':
2 כוסות , 2 שעונים, 2 צבעים וכד').

- 0% אינו מבצע התאמה
- 1% חוסר עקביות בהתאמה
- 2% התאמה חלקית עם דעיכה (מתאים כ- 50% מהפריטים)
- 3% התאמה כמעט מלאה (כ-75% מהפריטים) - הפרעות בקשב וריכוז.
- 4% מבצע התאמה זהה בעקביות .

.../.

הבנה שמיעתית

1. תגובה לרעש - הבדיקה מתבצעת ע"י השמעת גירויים שמיעתיים שונים (משרוקיות, מחיאת כף, מצילתיים וכד') תוך שימת לב לעוצמת הרעש, תדירות הצליל, ומס' הגירויים שעוררו תגובה. רפרטואר התגובות שאנו עשויים לקבל מן הנבדק הוא כפי שצוין בסעיפים השונים של מוטוריקה כללית.

- 0% לא מגיב לרעש.
- 1% מגיב לעיתים רחוקות.
- 2% חוסר עקביות בתגובות או איחור בולט או דעיכה בולטת.
- 3% מגיב ברוב המקרים, יתכן איחור בתגובה.
- 4% מגיב לגירויים שמיעתיים בעקביות.

2. תגובה לדיבור (לקול)

- 0% לא מגיב לקולות אנושיים כגון קריאת שמו, ברכה וכד' או לקולות אחרים בעלי משמעות כגון חיות או כלי מוסיקה (קסטות לאימון שמיעתי).
- 1% תגובות לעיתים רחוקות.
- 2% חוסר עקביות בתגובות או דעיכה בולטת.
- 3% מגיב ברוב המקרים, יתכן איחור בתגובה.
- 4% מגיב בעקביות.

3. ביצוע פקודות (מרכיב אחד)

יש לבדוק מינימום ביצוע של 3 פקודות שונות בזמן בדיקה אחד מתוך מגוון רחב יותר של פקודות שיורכב עבור כל חולה אינדיבידואלית בהתאם למצבו המוטורי-סנסורי. חשוב לשנות את סדר הצגת הפקודות בכל ניסיון.

- 0% אינו מבצע פקודות בעלות מרכיב אחד
- 1% מגיב לפקודות באופן פרסברטיבי, סטריוטיפי או ביזארי.
- 2% מבצע באופן לא עקבי פקודות פשוטות.
- 3% דעיכה בתגובות, הפסקה בתפקוד.
- 4% מבצע פקודות בעלות מרכיב אחד באופן עקבי.

.../.

4. <u>זיהוי פריטים</u>
החומרים לבדיקה: חפצים ותמונות .
בשלב ראשון יש להציג לנבדק בשדה ראיתו (או ע"י שימוש) 2 חפצים שונים לגמרי מבחינת קריטריוני המיון שלהם (כגון: כדור, בקבוק). בשלב הבא ניתן להציג לחולה 2 חפצים דומים לפחות בשני קריטריוני מיון
(כגון: כדור, תפוז) . דבר דומה יעשה עם 2 תמונות. בשלבים מתקדמים יותר אפשר להגדיל את מספר הגירויים (חפצים או תמונות) ואף להציג מילים כתובות ע"פי אותו סדר קושי עולה .

- 0% לא מזהה .
- 1% זיהוי מקרי או לעיתים רחוקות .
- 2% חוסר עקביות בזיהוי או דעיכה בולטת.
- 3% מזהה ברוב המקרים, יתכן איחור בתגובה או הפסקות בזיהוי .
- 4% מזהה בעקביות .

5. <u>ביצוע פקודות (שני מרכיבים)</u>
יש להתייחס לאותן הנחיות שהוזכרו בסעיף 3 בתחום בדיקה זה.

- 0% לא מבצע פקודות בעלות שני מרכיבים .
- 1% מבצע רק חלק מהפקודות תוך שינוי תוכן הפקודה .
- 2% מבצע באופן לא עקבי רק חלק מהפקודות (התחלה או סוף).
- 3% מסוגל לבצע פקודות בעלות שני מרכיבים ברוב המקרים, יתכנו הפסקות, איחורים או תנודות בביצוע .
- 4% מבצע פקודות בעלות שני מרכיבים באופן עקבי .

.../.

תקשורת

בתחום בדיקה זה קיימת אפשרות בחירה לבודק בין תקשורת מילולית לבין חליפית ע"פי מצבו האינדיבידואלי הסנסורי-מוטורי של החולה .
על הבודק לבטל על טופס הבדיקה את החלק שאינו בשימוש. יש להניח שחולים בשלב בדיקה זה הינם במצב הכרתי כלשהו.

<u>תקשורת מילולית</u>

1. <u>שימוש באיברי הדיבור</u> - ניסיון העברת בדיקת דיסארטריה/אפרקסיה

- 0% אין תנועתיות של איברי הדיבור,טריסמוס, אנארתריה. אין אין תנועות בחיקוי.
- 1% מגבלות קשות בתנועתיות איברי הדיבור, חיקוי לקוי .
- 2% הגבלות בתנועתיות של חלק מאיברי הדיבור, חיקוי חלקי או לא עקבי.
- 3% קושי בהתארגנות מוטורית/קואורדינציה
- 4% תנועתיות איברי הדיבור טובה . אין אפרקסיה BF .

2. <u>דיבור בסיסי</u>

- 0% אין הפקת קול
- 1% הפקת קולות שונים לעיתים רחוקות (אנחות, המהום, מלמול) ללא משמעות גלויה. יתכן מאמץ הפקתי על רקע נשימתי או דיספרקסי .
- 2% הפקת קולות שונים לא עקביים לצורך העברת מסר כלשהו (ניסיונות בסיסיים לתקשורת מילולית).
- 3% הפקת צלילים מוסכמים, הברות שלמות בעלות משמעות גלויה עם תופעות של דעיכה או הפסקות בתפקוד .
- 4% הפקה עקבית של מילים בודדות או צלילים בעלי משמעות לצורך תקשורת .

3. <u>היגוי</u>

- 0% אין ניסיונות היגוי
- 1% הזזת איברי הדיבור תוך חיפוש אינגרמות או מאמץ הפקתי על רקע דיספרקסי או התארגנותי (עם או בלי הפקת קול)(disuse)
- 2% דיבור מרוח מאוד או הפקות ג'רגוניות , מובנות דיבור ירודה ביותר .
- 3% שיבושי היגוי, פרפזיות ליטרליות, דיבור מרוח, מובנות דיבור ירודה .
- 4% היגוי תקין .

.../.

4. <u>קצב/רצף</u>
ההתייחסות בפרמטר זה הינה אך ורק לאלמנטים הסופרה-סגמנטלים של הדיבור .

- 0% אין שליטה על פרוזודיה, מהירות או תהודה.
- 1% שליטה מינימלית על מהירות העברת המסר, פרוזודיה מינימלית ו/או תהודה לקויה .
- 2% הפרעות קשות באינטונציה (מונוטוניה) ו/או במהירות הדיבור (טכילליה, ברדילליה) ו/או הפרעות קשות בתהודה (אפיות, היפונזליות) .
- 3% הפרעות באינטונציה (מונוטוניות) , ו/או במהירות הדיבור (טכילליה, ברדילליה) ו/או הפרעות בתהודה (אפיות, היפונזליות) .
- 4% אין הפרעות או הפרעות קלות באינטונציה, במהירות ו/או תהודה .

5. <u>איכות ההבעה</u>

- 0% הדיבור אינו מובן או אינו לעניין .
- 1% מעביר מסר מצומצם בלבד, מובנות הדיבור ירודה ביותר .
- 2% איכות העברת המסר לא עקבית, ירודה, תנודות בהתנהגות המילולית, יתכן על רקע דיסארטרי/אפרקטי/דמנטי/פרונטלי.
- 3% מסרים מובנים באופן חלקי, פרסברציות, פרפזיות, Slurring וכו' .
- 4% ניתן להבין את רוב המסר המועבר .

.../.

תקשורת חליפית

התאמת תקשורת חליפית מתבצעת בתיאום עם המחלקה לריפוי בעיסוק ע"פי צרכיו האינדיווידואליים של כל חולה.

1. צורך בעזרה חיצונית

- 0% איננו מתקשר גם עם עזרה חיצונית.
- 1% זקוק לעזרה חיצונית מרובה כגון: כיוון, הובלה והחזקת היד, הראש או כל איבר אחר המשתתף בתקשורת החליפית.
- 2% זקוק לעזרה חיצונית בחלק מהתפקוד או מהזמן. ברגע שמופסק התפקוד זקוק שוב לעזרה החיצונית.
- 3% מסוגל להשתמש במכשור לאחר הכנתו הראשונית עם הפסקות בתפקוד או דעיכה.
- 4% משתמש בתקשורת החליפית עם עזרה חיצונית מינימלית. (הנחת המכשור במקום המתאים לצורך ההפעלה או הכנת המכשור לשימוש).

2. שימוש באיברי גוף

פרמטר זה מתקשר ישירות לתחום הבדיקה של המוטוריקה הכללית מבחינת יכולתו המוטורית של החולה. יש לציין בדף הבדיקה באילו חלקי גוף יכול להשתמש החולה לצורך התקשורת החליפית. כמו כן יש לדרג את פונקציונאליות ההפעלה מ- 0% עד 4% ע"פי המפתח

3. יזימה

- 0% איננו יוזם שימוש בתקשורת החליפית.
- 1% יוזם לעיתים רחוקות בלבד.
- 2% חוסר עקביות ביזימה.
- 3% דעיכה ביזימה תוך כדי השימוש בתקשורת החליפית.
- 4% יזימה יפה לשימוש בתקשורת החליפית.

4. מהירות

- 0% מהירות התפקוד לא מאפשרת שימוש בתקשורת החליפית, יתכן ותופיע מהירות מופרזת (אימפולסיביות) או מאידך איטיות מוגזמת שמונעת יעילות התפקוד.
- 1% שליטה מעטה על מהירות התפקוד.
- 2% שליטה לא עקבית במהירות התפקוד.
- 3% דעיכה בקצב וברצף השימוש בתקשורת החליפית.
- 4% קצב ורצף יעילים.

.../.

5. איכות העברת המסר

- 0% אינו מסוגל להעביר מסר .
- 1% מעביר מסר מינימלי שקשה להבינו . הפרעות קשות בריכוז .
- 2% מעביר מסר חלקי, לא גמור, יתכנו הפסקות בתפקוד וניתוקים.
- 3% מעביר מסר חלקי שניתן להבינו או שמאפשר להסיק את המידע שאליו התכוון .
- 4% מעביר מסרים מובנים .
