# Supplementary material for: Reliability and Validity of the Lowenstein Communication Scale
Source: Neurol Int. 2025 Jul 29;17(8):116. doi: 10.3390/neurolint17080116 (PMC12388834; doi:10.3390/neurolint17080116)
Supplement: Supplementary file 1 [file neurolint-17-00116-s001.zip › Table S2 070725.pdf]

Table S2. Individual patient data

Serial number	Patient	Age	Sex	TBI or NTBI	NTBI etiology	Aphasia suspected	Time to admission to ICCR (days)	Consciousness state at admission to ICCR	Length of stay in ICCR (days)	Evaluation 1	Evaluation 2
1	1	27	Female	NTBI	Intracerebral haemorrhage, BH	No	37	MCS	66	Performed	Not performed
2	2	52	Male	NTBI	Intracerebral haemorrhage (Spontaneous), LH	No	41	UWS	62	Performed	Not performed
3	3	22	Male	TBI		No	31	MCS	104	Performed	Not performed
4	4	24	Female	TBI		No	32	UWS	19	Performed	Not performed
5	5	60	Male	TBI		No	50	MCS	49	Performed	Performed
6	6	43	Male	TBI		No	41	UWS	104	Performed	Not performed
7	7	20	Male	TBI		No	32	MCS	72	Performed	Performed
8	8	40	Male	NTBI	AVM PICA, BH	No	27	UWS	101	Performed	Not performed
9	9	70	Female	TBI		No	52	UWS	65	Not performed	Performed
10	10	65	Male	TBI		No	81	UWS	94	Performed	Not performed
11	11	59	Male	NTBI	Intracerebral haemorrhage, RH basal ganglia	No	39	UWS	66	Performed	Not performed
12	12	52	Male	NTBI	Anoxic brain damage	No	70	UWS	156	Performed	Not performed
13	13	63	Male	TBI		No	32	UWS	104	Performed	Not performed
14	14	54	Female	NTBI	Cerebral aneurism, basilar,LH	No	103	UWS	119	Performed	Not performed
15	15	41	Male	NTBI	Intracerebral haemorrhage (Spontaneous), RH	No	26	MCS	27	Performed	Not performed

16	16	48	Male	TBI			38	MCS	38	Performed	Performed
17	17	20	Male	NTBI	Anoxic brain damage	No	73	UWS	86	Performed	Performed
18	18	55	Female	NTBI	Cerebral aneurism, BH	No	36	UWS	60	Performed	Performed
19	19	66	Female	NTBI	Cerebral aneurism, RH	No	29	UWS	59	Performed	Performed
20	20	69	Male	NTBI	CVA, LH	Yes	27	MCS	48	Performed	Performed
21	21	54	Male	TBI		No	50	MCS	80	Performed	Performed
22	22	53	Male	TBI		No	32	UWS	55	Performed	Performed
23	23	55	Male	TBI		No	34	UWS	44	Performed	Performed
24	26	71	Female	NTBI	Intracerebral haemorrhage, RH	No	30	MCS	58	Performed	Not performed
25	27	46	Male	TBI		No	46	MCS	72	Performed	Performed
26	28	54	Female	NTBI	Aneurism of brain, RH	No	96	MCS	78	Performed	Not performed
27	29	56	Male	NTBI	Intracerebral haemorrhage, LH basal ganglia	No	32	MCS	47	Performed	Performed
28	30	29	Male	TBI		No	59	MCS	79	Performed	Not performed
29	31	42	Female	TBI		No	34	MCS	71	Performed	Not performed
30	32	52	Male	NTBI	Intracerebral haemorrhage (Spontaneous), LH basal ganglia	No	72	MCS	41	Performed	Not performed
31	33	59	Male	TBI		No	34	UWS	149	Performed	Not performed
32	34	47	Male	TBI		No	44	UWS	167	Performed	Performed
33	35	59	Male	TBI		No	39	UWS	173	Performed	Performed
34	36	33	Male	TBI		No	40	MCS	59	Performed	Performed
35	37	24	Female	TBI		No	75	UWS	131	Performed	Performed
36	38	27	Female	TBI		No	26	UWS	43	Performed	Not performed
37	40	45	Female	TBI		No	41	UWS	31	Performed	Not performed

38	41	53	Female	NTBI	Aneurism of brain, BH	No	88	MCS	97	Performed	Not performed
39	42	39	Male	TBI		No	35	UWS	167	Performed	Performed
40	43	37	Male	NTBI	Anoxic brain damage, BH	No	44	MCS	111	Performed	Not performed
41	44	63	Male	NTBI	Intracerebral haemorrhage, BH	No	41	UWS	82	Performed	Performed

Abbreviations : ICCR=intensive care for consciousness rehabilitation, TBI=traumatic brain injury, CVA=cerebrovascular accident, AVM=Arteriovenous Malformation, RH=right hemisphere, LH=left hemisphere, BH=both hemispheres, NTBI= non-traumatic brain injury, UWS=unresponsive wakefulness syndrome, MCS=minimally conscious state
